# A New Way for Beta Cell Neogenesis: Transdifferentiation from Alpha Cells Induced by Glucagon-Like Peptide 1

**DOI:** 10.1155/2019/2583047

**Published:** 2019-03-13

**Authors:** Zhen Zhang, Yinghui Hu, Ningning Xu, Wenjun Zhou, Lei Yang, Rongping Chen, Rui Yang, Jia Sun, Hong Chen

**Affiliations:** ^1^Department of Endocrinology, Zhujiang Hospital, Southern Medical University, Guangzhou, China; ^2^Department of International Medical Center, The First Affiliated Hospital of Xi'an Jiaotong University, Shangxi, China; ^3^Department of Nephrology, Zhujiang Hospital, Southern Medical University, Guangzhou, China

## Abstract

Recent studies showed that alpha cells, especially immature cells and proalpha cells, might be the precursors of beta cells. Exposure to glucagon-like peptide 1 (GLP1) can ameliorate hyperglycemia in diabetic mice and restore the beta cell mass. In the present study, we adopted single high-dose (60 mg/kg, i.p.) streptozotocin (STZ) to model diabetes mellitus (DM) and randomly assigned short-tail (SD) rats to a normal group, a diabetic group, GLP1 groups (50 *μ*g/kg, 100 *μ*g/kg, and 200 *μ*g/kg), a GLP1 (200 *μ*g/kg) with exendin (9-39) group, and a GLP1 with LY294002 group. We found that the pancreatic insulin-glucagon-positive cell populations increased according to the increase in GLP1 exposure. By contrast, no insulin-amylase-positive cell populations or insulin/pan-cytokeratin cells were observed in the pancreatic sections. The GLP1 receptor antagonist exendin (9-39) and the phosphatidylinositol-4,5-bisphosphate 3-kinase (PI3K) family inhibitor LY294002 not only suppressed protein kinase B (*Akt*), pancreatic and duodenal homeobox 1 (*Pdx1*), forkhead box O 1 (*FoxO1*), and mast cell function-associated antigen A (*MafA*) mRNA expression but also increased *MAFB* expression. We concluded that treatment with GLP1 might result in beta cell neogenesis by promoting the transdifferentiation of alpha cells but not by pancreatic acinar cells, ductal cells, or the self-replication of beta cells. The regulation on the GLP1 receptor and its downstream transcription factor PI3K/AKT/FOXO1 pathway, which causes increased pancreatic and duodenal homeobox 1 (*Pdx1*) and *MafA* mRNA expression but causes decreased *MAFB* expression, may be the mechanism involved in this process.

## 1. Introduction

The impoverishment or functional decline in pancreatic beta cells is the main cause of all forms of diabetes [1]. Currently, therapy for diabetes comprises drug therapy or pancreatic islet transplantation. The influences of the environment and other exogenous factors mean that a transplanted pancreas does not play a good role in regulating blood glucose. Thus, endogenous proliferation of functional islet beta cells has become a focus of research attention [2]. Pancreatic exocrine cells (pancreatic ductal cells and pancreatic acinar cells) and pancreatic cells (liver cells) can be transformed into islet cells [3]. In experimental transgenic models of diphtheria toxin- (DT-) induced acute selective near-total beta cell ablation, researchers observed beta cell regeneration. They used lineage tracing to label the glucagon-producing alpha cells and found that beta cell regeneration was largely derived from alpha cells before beta cell ablation, revealing previously unrecognized pancreatic cell plasticity [4]. Other studies observed a large number of glucagon-insulin-positive cells with extreme beta cell loss induced by streptozotocin (STZ), which is considered an important process to transform alpha cells into beta cells [5, 6]. Such spontaneous conversion of adult pancreatic alpha cells into beta cells could be harnessed to treat diabetes.

Glucagon-like peptide 1 (GLP1) is a gut-derived hormone secreted by intestinal L cells in response to food intake. GLP1 has been a prospective target for type 2 diabetes therapy [7]. Numerous studies have shown that infusion of GLP1 can efficiently ameliorate hyperglycemia in diabetic models. Animal models demonstrated increasing and restored beta cell mass via beta cell regeneration, proliferation, and neogenesis after GLP1 administration [8]. Other studies showed that GLP1 acts mainly by activating GLP1 receptors, which upregulates the levels of pancreatic and duodenal homeobox 1 (PDX1) through the phosphatidylinositol-4,5-bisphosphate 3-kinase (PI3K)/AKT kinase (AKT) pathway. PDX1, known as a master regulator of the beta cell phenotype, plays a prominent role as an activator of genes essential for beta cell identity, along with the suppression of alpha cell identity [9, 10]. However, it remains unknown whether the augmentation of beta cell mass induced by GLP1 acts, at least in part, through transdifferentiation from alpha cells within the pancreas.

Therefore, the present study was aimed at investigating whether GLP1 could promote the regeneration of beta cells by the endogenous neogenesis of beta cells from the transdifferentiation of alpha cells in rat pancreatic islets and its possible mechanism.

## 2. Materials and Methods

### 2.1. Animals and Treatments

Sixty specific pathogen-free (SPF) level male Sprague-Dawley (SD) rats at eight to ten weeks old with a weight of 180–220 g were purchased from the Laboratory Animal Center of the Southern Medical University. The rats were housed in groups with an artificial 12 h dark-light cycle and with free access to food and water. The animals were treated by intraperitoneal injection with 60 mg/kg STZ (Sigma-Aldrich, St. Louis, MO, USA) dissolved in 50 mM citrate buffer (pH 4.5). Blood glucose levels, body weights, and diabetes incidence were monitored weekly. Only rats with a blood glucose level greater than 28 mmol/L (measured after 72 hours of STZ injection) were selected for the experiments [11]. These rats (*n* = 60) were divided into a normal group (*n* = 6); a diabetic group (*n* = 9); GLP1 groups treated with subcutaneous injections of GLP1 50 *μ*g/kg/12 h (*n* = 9), 100 *μ*g/kg/12 h (*n* = 9), or 200 *μ*g/kg/12 h (*n* = 9); a GLP1 (200 *μ*g/kg) with exendin (9-39) group (*n* = 9); and a GLP1 with LY294002 group (*n* = 9) for 12 weeks [12]. Numerous studies have shown that infusion of GLP1 can efficiently ameliorate hyperglycemia in diabetic models [13, 14]. GLP1 has been shown to increase beta cell mass, based on *in vitro* studies. It has also been shown to increase beta cell mass in animal models through beta cell regeneration, proliferation, and neogenesis and through the inhibition of apoptosis [15]. Miao et al. [8] indicated that treatment with 100 nM liraglutide (a GLP1 derivative) for 24–72 h promoted cell proliferation in the presence of 30 mM glucose, and the liraglutide increased beta cell viability at an optimum concentration of 100 nM in the presence of 11.1 or 30 mM glucose. After confirming previous evidence that GLP1 reduced blood glucose level and body weight, we chose the GLP1 concentrations utilized in the present study. All animal experiments were approved by the Committee on Animal Experimentation of Southern Medical University, Guangzhou, China and performed in compliance with the university's Guidelines for the Care and Use of Laboratory Animals. The Ethics Committee approval number is L2015106.

### 2.2. Antibodies and Reagents

GLP1 was obtained from Novo Nordisk A/S (Bagsvaerd, Denmark). Insulin and the C-peptide assay (enzyme-linked immunosorbent assay (ELISA)) kit were from Mercodia (Uppsala, Sweden). The anti-insulin mouse monoclonal antibody (SAB4200691) and anti-forkhead box O 1 (FOXO1) antibody (AV32107) were from Sigma-Aldrich. The anti-glucagon antibody (2760S) was from CST Biological Reagents Company Limited (Shanghai, China). The anti-mast cell function-associated antigen B (MAFB) antibody (DF8895) and anti-PDX1 antibody (DF7170) were from Affinity Biosciences (Cincinnati, OH, USA). The anti-neurogenin 3 (NGN3) antibody (GTX60254) was from GeneTex Inc. (Irvine, CA, USA). The primary antibodies against MAFA (sc-390491), pan-Cytokeratin (sc-8018), AKT1/2/3 (sc-81434), exendin (9-39) (SC-364387), and amylase were from Santa Cruz Biotechnology Inc. (Santa Cruz, CA, USA). The PI3K inhibitor LY294002 hydrochloride was also from Sigma-Aldrich.

### 2.3. Measurement of Metabolic and Biochemical Parameters

Body weight was measured weekly in each group for the entire duration of the study. The fasting glucose level (12-hour food deprivation) was measured using a glucometer (FreeStyle Optium; Abbot Laboratories, Italy) in blood taken weekly from the tail vein from rats in each experimental group. Plasma insulin concentrations and C-peptide levels were determined using ELISA assays after the intervention. At the end of the study period, all rats were anesthetized using pentobarbital (0.1 mg/g intraperitoneal injection) and sacrificed.

### 2.4. Immunofluorescence Analysis and Immunohistochemistry

After double-labeling immunofluorescence, areas labeled for insulin (green) and glucagon (red) in pancreatic islets were assessed, as described previously [16]. Pancreatic sections (6 *μ*m thick) of normal and diabetic rats were deparaffinized with xylene and rehydrated with descending concentrations of ethanol. Antigen retrieval was then conducted by boiling the slides in citrate buffer, followed by gradual cooling. The sections were treated with a blocking agent (0.5% bovine serum albumin) for 45 minutes at room temperature after washing in phosphate-buffered saline (PBS), and then they were incubated overnight at 4°C with anti-insulin antibodies (1 : 1000 dilution) followed by the corresponding fluorescein isothiocyanate- (FITC-) conjugated anti-guinea pig secondary antibody after washing in PBS. The sections were then incubated overnight at 4°C with anti-glucagon, amylase, and pan-Cytokeratin antibodies (prediluted) followed by the anti-rabbit tetramethylrhodamine- (TRITC-) conjugated secondary antibody. Finally, the sections were mounted using Immu-Mount® (Thermo Electron Corporation, Waltham, MA, USA) and viewed with a Carl Zeiss fluorescent microscope.

Proliferation of pancreas islets was analyzed by immunohistochemical staining of proliferating cell nuclear antigen (PCNA) using anti-PCNA antibodies. The slides were incubated with primary antibodies for 1 hour at room temperature. After washing, secondary antibodies (1 : 500, biotin-conjugated goat anti-rabbit IgG) were applied for 30 min at room temperature. The number of PCNA-positive cells in each islet section was counted under a microscope. Three islets were randomly selected for analysis in each rat [17].

### 2.5. RNA Extraction and Quantitative Real-Time Reverse Transcriptase Polymerase Chain Reaction (qRT-PCR)

Total RNA was isolated from the pancreatic tissue mentioned above using the TRIzol reagent (Takara Bio Inc., Dalian, China) according to the manufacturer's instructions, after which the RNA quantity and purity were evaluated using a model ND-2000 apparatus (Thermo Fisher Scientific NanoDrop 2000, Waltham, MA, USA). The integrity of the RNA was confirmed by agarose-formaldehyde gel electrophoresis. Using a cDNA Reverse Transcription Kit (Promega Corporation, Madison, WI, USA), first-strand cDNA was synthesized from individual samples in 20 *μ*L reactions using 200 ng of total RNA, following the manufacturer's instructions. The integrity of the cDNA was confirmed by amplifying the *Atcb* (beta-actin) gene. Real-time PCR was conducted using a LightCycler 96 (Roche Applied Science, Rotkreuz, Switzerland) employing SYBR Green I as the dsDNA-specific binding dye for continuous fluorescence monitoring. The reverse transcription protocol was as follows: 5 min at 95°C, followed by 45 cycles of 15 s at 95°C, 15 s at 58°C, and 15 s at 72°C. The primers were synthesized by Takara Bio Inc. (Table 1).

### 2.6. Western Blotting Analysis

Total protein extracts to detect PDX1, MAFA, MAFB, AKT, and FOXO1 levels were obtained as previously described [12], pancreatic tissues were homogenized in lysis buffer (0.25 mol/L sucrose, 1 mmol/L EDTA) supplemented with protease inhibitor cocktail (Sigma-Aldrich) and phosphatase inhibitors (Sigma-Aldrich), and then the samples were centrifuged for 10 min at 16,000 × *g*. Protein concentrations were determined using a BCA Protein Assay Kit (Pierce Biotechnology, Rockford, IL, USA). Subsequently, the protein samples (20 *μ*g) were separated on a 10% SDS-PAGE gel at 80 V (stacking gel) and 100 V (separating gel) and transferred onto nitrocellulose membranes. The membranes were blocked with PBS-Tween (PBS-T) containing 5% nonfat milk for 1 hour at room temperature. After blocking nonspecific binding, the membranes were incubated overnight at 4°C with mouse monoclonal primary antibodies against PDX1 (diluted 1 : 4000), MAFA (diluted 1 : 1000), MAFB (diluted 1 : 800), AKT (diluted 1 : 800), FOXO1 (diluted 1 : 1000), and glyceraldehyde-3-phosphate dehydrogenase (GAPDH) (diluted 1 : 10,000). Subsequently, the membranes were washed with PBS-T three times and incubated for 1 hour with a goat anti-mouse IgG antibody conjugated to horseradish peroxidase (HRP) (1 : 1000; catalog no. sc-2031; Santa Cruz Biotechnology Inc.). Lastly, the membranes were washed three times with PBS-T, and the immunoreactive proteins were visualized using an enhanced chemiluminescence- (ECL-) detection system (Beyotime Institute of Biotechnology, Jiangsu, China).

### 2.7. Statistical Analysis

All data are expressed as the means ± standard error of the mean (SEM). Statistical analysis was performed using Student's *t-*test and one-way analysis of variance (ANOVA), followed by a post hoc Fisher protected least significant difference (Fisher PLSD) test. A value of *P* < 0.05 was considered statistically significant. Statistical analyses were conducted using SPSS 16.0 software (IBM Corp., Armonk, NY, USA).

## 3. Results

### 3.1. Establishment of an Extreme Beta Cell Loss Rat Model Induced by STZ

**Figure 1:**
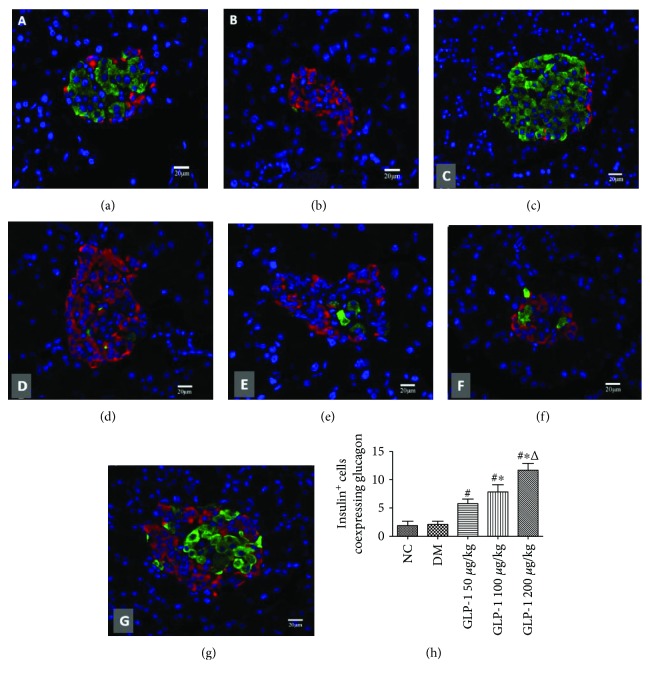
GLP1 reversed STZ-induced compromised *β* cell identity by inducing *α* cell transdifferentiation into *β* cells. Diabetic groups received an intravenous injection with STZ (35 mg/kg). Where indicated, GLP1 (50, 100, or 200 *μ*g/kg per rat) was also administered. (a–g) Confocal images of pancreatic islet sections double-labeled via immunofluorescence for insulin and glucagon. Insulin^+^ cells' immunoreactivity was distinguished by green fluorescence, while glucagon^+^ cells were identified using FITC immunofluorescence (red) in the same slices. Scale bar = 20 *μ*m. (h) Ratio of insulin^+^-glucagon^+^ cells to insulin^+^-glucagon^−^ cells. Data are presented as the mean ± SEM (*n* ≥ 6 rats per group), *P* < 0.01, one-way ANOVA. GLP1, glucagon-like peptide 1; STZ, streptozotocin; FITC, fluorescein isothiocyanate; SEM, standard error of the mean; ANOVA, analysis of variance; NC, negative control; DM, diabetes mellitus.

Thorel et al. demonstrated that after extreme beta cell loss, a proportion of new beta cells transdifferentiated from alpha cells via a bihormonal glucagon/insulin (Gcg/Ins) coexpressing transitional state [4]. Thus, the present study was conducted using a single high dose of STZ (60 mg/kg) injection to induce a near-total beta cell ablation diabetic rat model to investigate the role of GLP1 in the neogenesis of beta cells. The results of double-labeled immunofluorescence in rat pancreatic slices showed that the pancreatic islets of the SD rats were significantly reduced, and the results also showed 95% insulin-positive beta cell ablation **(**Figures 1(a) and 1(b)**)**. The islets showed the serious structural damage of beta cells, with alpha cell invasion into the center of the islets; very few residual beta cells were found scattered around the alpha cells. To confirm that the endogenous beta cell regeneration induced by GLP1 mainly occurs by transdifferentiation from alpha cells, we used double-labeled immunofluorescence technology to trace the development of different types of islet cells. The results demonstrated that after intervention with GLP1, insulin-glucagon-positive cells appeared in the islets of the pancreas in the diabetic rats and increased in a dose-dependent and statistically significant manner (*P* < 0.05) compared with those in the normal control group and the diabetic model group (Figures 1(c)–1(h)).

### 3.2. GLP1 Protects Beta Cells against Glucose Toxicity in Diabetic Rats

**Figure 2:**
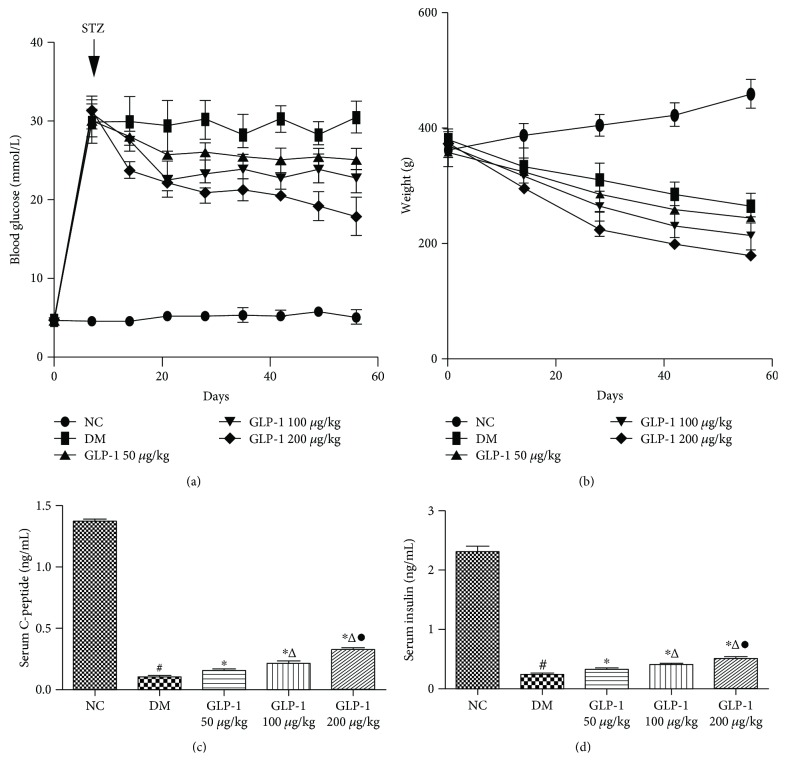
Fasting blood glucose, weight, and serum C-peptide, together with insulin levels, in rats from different treatment groups. (a). Profiles of 60-day fasting blood glucose and weight. (b). Serum C-peptide and insulin levels after 60 days. Data are presented as the mean ± SEM (*n* ≥ 6 rats per group), *P* < 0.05, one-way ANOVA. GLP1, glucagon-like peptide 1; SEM, standard error of the mean; ANOVA, analysis of variance; NC, negative control; DM, diabetes mellitus.

After confirming the previous observation [4] that GLP1 reduced blood glucose level and body weight (data not shown), we investigated its effects in STZ-DM rats (the blood glucose level and weight were detected every week). After GLP1 intervention, insulin and C-peptide were measured using ELISA, and the number of endocrine cells was analyzed using double immunofluorescence. As expected, we found that GLP1 induced a significant decrease in the weight and blood glucose level in a dose-dependent manner compared with that in the control group (Figures 2(a) and 2(b)). However, even when treated with the maximum dose of GLP1 (200 *μ*g/kg), the blood glucose level of the diabetic rats stayed at a high level (>19 mmol/L). Meanwhile, GLP1 resulted in a significant increase in insulin and C-peptide levels (Figures 2(c) and 2(d)).

### 3.3. Evidence for Endogenous Beta Cell Regeneration from Alpha Cells in Rat Pancreatic Islets Induced by GLP1

**Figure 3:**
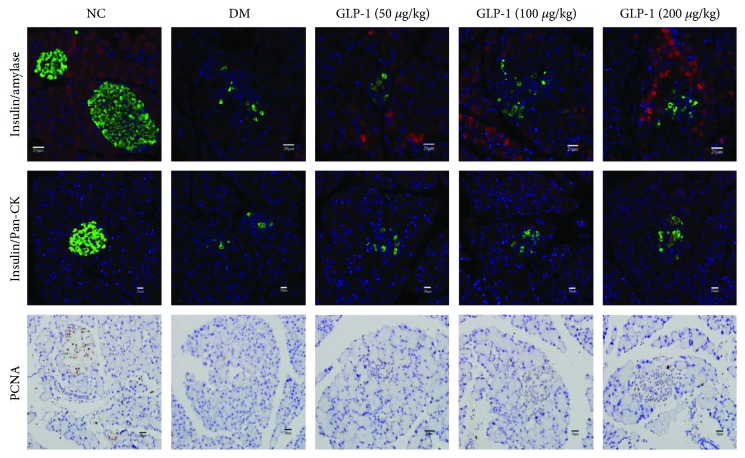
GLP1 did not promote pancreatic acinar or ductal cells to transform into islet *β* cells. (a) Pancreatic frozen sections were immunolabeled for insulin (green), amylase, or Pan-CK (red) as indicated. (b) PCNA immunohistochemical staining for pancreatic paraffin-embedded slices. Pan-CK; pan-cytokeratin; GLP1, glucagon-like peptide 1; NC, negative control; DM, diabetes mellitus; PCNA, proliferating cell nuclear antigen.

Under specific conditions, the rodent pancreas is capable of regeneration and cell plasticity. Many studies have shown that pancreatic epithelial cells (ductal, acinar, alpha, and beta cells) are potential alternatives to pluripotent stem cells because of the tremendous differentiation capacities of beta cells and their lower safety concerns [2]. The results demonstrated that after intervention with GLP1, insulin-glucagon-positive cells are at an intermediate state of transformation from alpha cells to beta cells. By contrast, no insulin-amylase-positive and insulin-pan-CK-positive cells were found in pancreases of all groups (Figure 3) indicating that subcutaneous injection of GLP1 did not promote pancreatic acinar cells or ductal cells of the STZ-induced diabetic rats to transform into islet beta cells. We also used PCNA immunohistochemistry to assess the proliferation of rat pancreatic beta cells after GLP1 intervention. We found that the region of PCNA-stained cells was coincident with the distribution of islet beta cells in the normal rat pancreas, whereas in the diabetic model group and the GLP1 groups, the PCNA-stained cells were mainly located in the region containing alpha cells, and there were no significant differences in PCNA-stained cells between the diabetic model group and the GLP1 groups (*P* < 0.05) (Figure 3). These results indicated that the endogenous beta cell regeneration and increase in beta cell number induced by GLP1 did not occur by the replication of beta cells but mainly from alpha cell transdifferentiation.

### 3.4. Effect of GLP1 Treatment on Expressions of the Key Genes Related to Beta Cell Development

**Figure 4:**
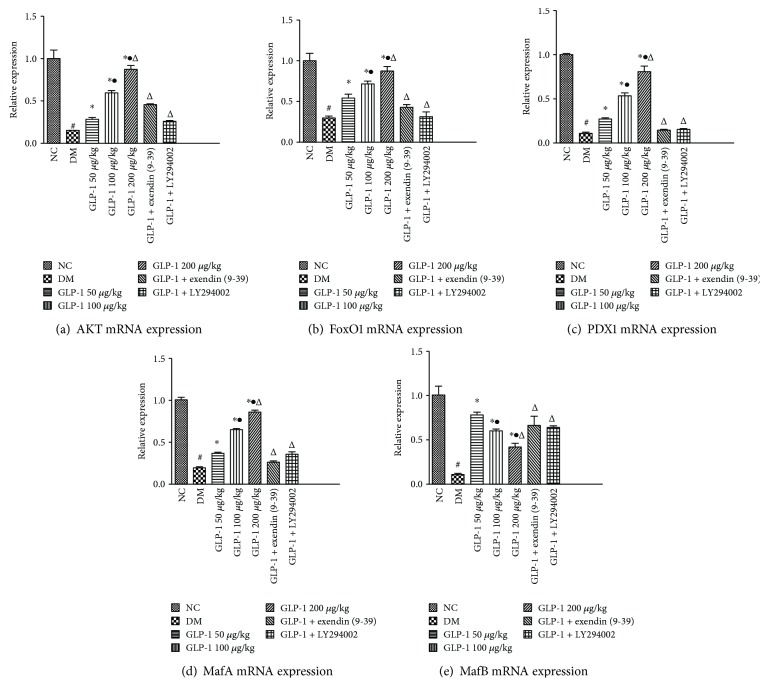
Quantitative real-time reverse transcription PCR (qRT-PCR) analyses for markers of *β* cell identity genes (*Akt*, *FoxO1*, *Pdx1*, *MafA*, and *MafB*) in rat islets. Data are presented as the mean ± SEM (^#^*P* < 0.05*vs*. NC; ^∗^*P* < 0.05*vs*. DM; ^•^*P* < 0.05*vs*. GLP1 50 *μ*g/kg; and ^Δ^*P* < 0.05*vs*. GLP1 100 *μ*g/kg). GLP1, glucagon-like peptide 1; NC, negative control; DM, diabetes mellitus; SEM, standard error of the mean; Akt, AKT kinase; FoxO1, forkhead box O 1; Pdx1, pancreatic and duodenal homeobox 1; MafA, MAF BZIP transcription factor A; MafB, MAF BZIP transcription factor B.

**Figure 5:**
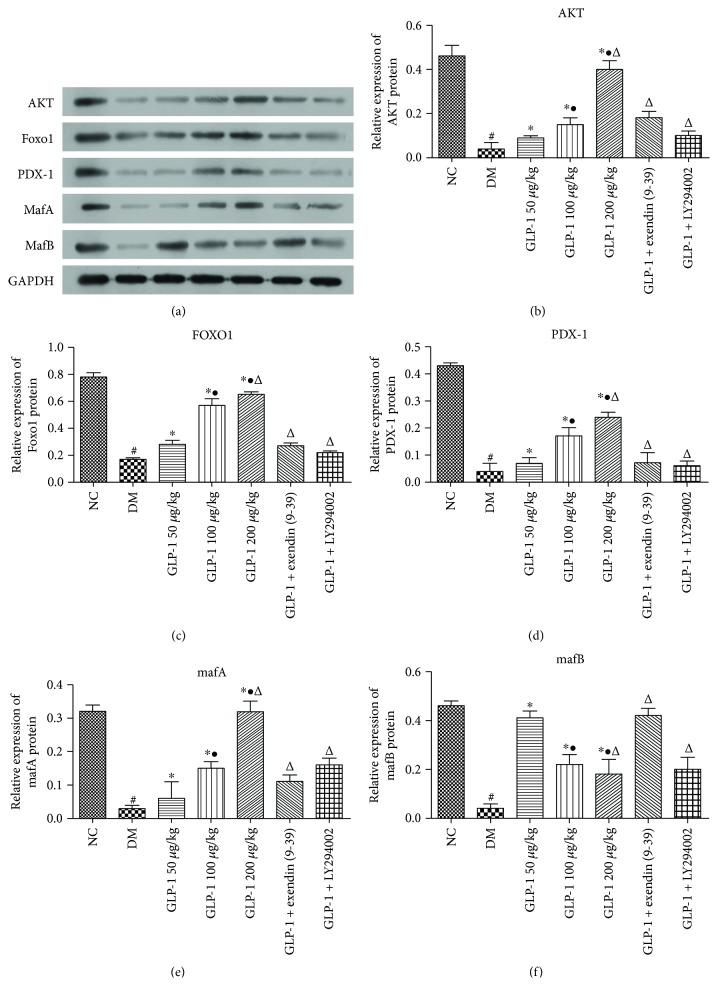
Western blotting analysis for Akt, FoxO1, Pdx1, MafA, and MafB in rat islets. GAPDH was used as a loading control (^#^*P* < 0.05*vs*. NC; ^∗^*P* < 0.05*vs*. DM; ^•^*P* < 0.05*vs*. GLP1 50 *μ*g/kg; and ^Δ^*P* < 0.05*vs*. GLP1 100 *μ*g/kg). GLP1, glucagon-like peptide 1; NC, negative control; DM, diabetes mellitus; Akt, AKT kinase; FoxO1, forkhead box O 1; Pdx1, pancreatic and duodenal homeobox 1; MafA, MAF BZIP transcription factor A; MafB, MAF BZIP transcription factor B; GAPDH, glyceraldehyde-3-phosphate dehydrogenase.

To investigate the possible mechanism by which GLP1 promotes alpha cells to transdifferentiate into beta cells, the mRNA and protein expressions of AKT, PDX1, MAFA, MAFB, and FOXO1 were detected by qRT-PCR and western blotting in rat pancreatic islets. The results showed that the expression levels of AKT, PDX1, FOXO1, MAFA, and MAFB in the diabetic model group were significantly lower than those in the normal group (*P* < 0.05). The addition of GLP1 induced significant increases in the expression levels of FOXO1, AKT, PDX1, and MAFA in a dose-dependent manner compared with those in the diabetic model group (*P* < 0.05) (Figures 4 and 5). The expression of MAFB showed an interesting expression pattern. At first, the injection of GLP1 (50 *μ*g/kg) also induced a marked increase compared with that in the diabetic model group. However, as the dose of GLP1 increased, the expression of MAFB decreased significantly in a dose-dependent manner. All of these effects could be attenuated using the GLP1 receptor blocker exendin (9-39) or the PI3K inhibitor LY294002 (*P* < 0.05) (Figures 4 and 5).

## 4. Discussion

Streptozotocin (STZ), an antibiotic extracted from *Streptomyces achromogenes*, can invade beta cells in rats, causing extreme DNA damage and cell death, resulting in hyperglycemia [18]. There are three main factors affecting the diabetic models induced by STZ, including the frequency, dose, and time of STZ injection [19]. High-fat diet/low-dose STZ injections induce models of type 2 diabetes mellitus (T2DM) [20], while two days of injection with STZ (45 mg/kg) into rats severely impaired insulin secretion and produced type 1 diabetes [21]. To exclude insulin resistance and self-immunity as possible confounders and to investigate the effects of GLP1 on rat pancreatic islets, we constructed severe insulin-deficient diabetic rat models induced by injecting a single dose of intraperitoneal STZ (60 mg/kg). The results showed that the pancreatic islets of the SD rats were significantly reduced, and the results also showed 95% insulin-positive beta cell ablation. We also observed that beta cells were seriously structurally damaged, with alpha cells invading into the center of the islets and very few residual beta cells being found scattered around the alpha cells. The blood glucose levels of diabetic rats stayed at a high level, with no spontaneous recovery, which implied that these rats are ideal models for the study of beta cell regeneration.

Similar to a previous study [22], we found that GLP1 induced a significant decrease of blood glucose and an increase of insulin and C-peptide levels in a dose-dependent manner compared with those in the control group. However, even when treated with GLP1 at the maximum dose of 200 *μ*g/kg, the blood glucose level of the diabetic rats stayed at a high level (>19 mmol/L), which indicated that in rats with type 1 diabetes mellitus, the effects of GLP1 on decreasing blood glucose level and improving beta cell function are limited. Meanwhile, we found that GLP1 induced a significant decrease in the weight of the rats in a dose-dependent manner compared with the control group, which agreed with previous studies [23] that showed that GLP1 might help to suppress appetite and induce body weight loss in the obese rats.

The appearance of insulin-glucagon-positive cells, which might be an intermediate state of transformation from alpha cells to beta cells, was found in the progenitor cells of the rat pancreatic islets [6]. Our results demonstrated that after intervention with GLP1, insulin-glucagon-positive cells appeared in the islets of the pancreases of diabetic rats and increased in a dose-dependent manner, which means that our observations are easily reconciled with the common view that GLP1 could promote beta cell neogenesis by promoting the transdifferentiation of alpha cells.

Hui et al. [24] observed that in *in vitro* studies, the GLP1 receptor agonist, exendin-4, and GLP1 promoted pancreatic acinar cells into insulin-producing cells via the PKC-MAPK signaling pathway. Bonner-Weir et al. [25] have provided evidence that the formation of islet-like cell clusters occurs after culturing ductal cells derived from human pancreas, and these cell clusters can secrete a small amount of insulin under stimulation by glucose. Gao et al. [26] used the bromodeoxyuridine (BrdU) marker to show that part of the pancreatic ductal cells transformed into islet endocrine cells, resulting in the alleviation of hyperglycemia in diabetic rats [27]. To determine the source of beta cell neogenesis, we used double-labeled immunofluorescence technology. Our discovery of the lack of insulin-amylase-positive and insulin-Pan-CK-positive cells in specimens of the pancreas of all groups indicated that the subcutaneous injection of GLP1 did not promote pancreatic acinar cells or ductal cells of STZ-induced diabetic rats to transform into islet beta cells.

Esteban et al. suggested that the replication of beta cells plays an important role in endogenous neogenesis of beta cells [28]. To test this hypothesis, we used PCNA immunohistochemistry to assess the proliferation of rat pancreatic beta cells. We found that the region of PCNA-stained cells was coincident with the distribution of islet beta cells in the normal rat pancreas (Figure 4), while in the diabetic model group and the GLP1 group, the cells were mainly located in the region showing alpha cell distribution, and there were no significant differences in PCNA-stained cells between the diabetic model group and the GLP1 group. These results indicated that the endogenous beta cell regeneration and increase in the number of beta cells induced by GLP1 was not derived from the replication of beta cells but was derived mainly from alpha cell transdifferentiation.

The MAF family is subdivided into two groups, according to their structure: the large and small MAF transcription factors [29–31]. In adult mammals, MAFA, which is essential in maintaining pancreatic islet structure and beta cell function, stimulates insulin gene expression and promotes insulin secretion [32]. When the insulin gene is transcribed, MAFA, as the insulin gene promoter, acts on the cis-regulatory element, termed RIPE3b/C1, located in the insulin gene promoter. The expression levels of transcription factors such as PDX1, Beta2, and NeuroD were reduced in rats displaying low expression of the *MafA* gene, and the number of cells and the secretion of insulin were reduced as well [33, 34]. MAFB is only expressed in mature alpha cells, and MAFA is a marker of mature beta cells. In the process of alpha cells' transformation into beta cells, the expression level of MAFB gradually changed from high to low, while that of MAFA showed the opposite trend [35].

The results showed that the expression levels of AKT, PDX1, FOXO1, MAFA, and MAFB in the diabetic model group was significantly lower than those in the normal group. The injection of GLP1 resulted in a dose-dependent increase in AKT, PDX1, FOXO1, and MAFA expression, but it resulted in a decrease in MAFB expression. From these results, we concluded that GLP1 promotes beta cell function but inhibits alpha cells. The GLP1 receptor antagonist exendin (9-39) and the PI3K family inhibitor LY294002 suppressed *Akt*, *Pdx1*, *FoxO1*, and *MafA* mRNA expression; however, *MAFB* expression increased, indicating that GLP1 enhances the reactivity of PDX1 and MAFA, which are markers of beta cells via the GLP1 receptor and its downstream pathway. In addition, these results suggested that GLP1 regulates the expression of pancreas-specific transcription factors by binding to the GLP1 receptor located on the membrane surface and activating the downstream PI3K-AKT-FOXO1 signaling pathway. The western blotting results were consistent with those of the qRT-PCR results, which led us to believe that GLP1 promotes the transformation from alpha cells to beta cells, and this positive action of GLP1 is mediated by the activation of the PI3K/AKT/FOXO1 pathway, which results in the upregulation of PDX1 and MAFA.

However, there were some limitations in our research. First, some researchers insist that insulin-glucagon-positive cells are an intermediate state of transformation from alpha cells to beta cells, whereas others suggest that insulin-glucagon-positive cells, which are immature pancreatic endocrine cells, tend to develop into alpha cells in the early development of the rat endocrine pancreas. In addition, MAFB is only expressed in mature alpha cells and PDX1 is a marker of mature beta cells. Thus PDX1/glucagon and MAFB/insulin double-positive cells are also an intermediate state in the transformation of alpha cells to beta cells [36]. If we conducted more thorough double-labeled immunofluorescence technology using insulin/MAFB and glucagon/PDX1, the outcomes would be more convincing. Second, our research was an experimental validation based on a hypothesis; therefore, we propose to construct *Pdx1* gene knockout animal models by genetic engineering and use them to confirm the role of PDX1 in the transformation of alpha cells into beta cells.

In recent years, research on promoting beta cell regeneration has provided new methods for the treatment of diabetes. Our findings indicated that GLP1 treatment might result in beta cell neogenesis by promoting the transdifferentiation of alpha cells. The regulation of the GLP1 receptor and its downstream transcription factor pathway (PI3K/AKT/FOXO1), which causes increased *Pdx1* and *MafA* mRNA expression, but decreased *MAFB* expression, may be the mechanism involved in this process. The results of the present study may be useful to study cellular and molecular mechanisms that regulate adult pancreatic differentiation and will increase our understanding of pancreatic diseases, such as cancer and diabetes.

## Figures and Tables

**Table 1:** List of primer sequences used for RT-PCR.

ID	Sequence (5′-3′)	Product length (bp)
*β*-Actin F	GGAGATTACTGCCCTGGCTCCTA	150
*β*-Actin R	GACTCATCGTACTCCTGCTTGCTG	
GLP1 F	TCGTGGCTGGATTGTTTGTA	143
GLP1 R	ATGGCGTTTGTCTTCGTTTAT	
AKT F	TCCCTTCCTTACAGCCCT	285
AKT R	TCCTTGATACCCTCCTTGC	
PDX1 F	GCCAGAGTTCAGTGCTAATCC	106
PDX1 R	TCCCTGTTCCAGCGTTCC	
MafA F	TTCAGCAAGGAGGAGGTCAT	117
MafA R	CTCGCTCTCCAGAATGTGC	
MafB F	GCTGGTGTCCATGTCCGT	245
MafB R	TGACCTTGTAGGCGTCTCTCT	
FOXO1 F	GTGGATGGTGAAGAGTGTGC	275
FOXO1 R	CGGACTGGAGAGATGCTTT	

## Data Availability

The data used to support the findings of this study are available from the corresponding author upon request.
